# Multi-Dimensional, Mesoscopic Monte Carlo Simulations of Inhomogeneous Reaction-Drift-Diffusion Systems on Graphics-Processing Units

**DOI:** 10.1371/journal.pone.0033384

**Published:** 2012-04-10

**Authors:** Matthias Vigelius, Bernd Meyer

**Affiliations:** FIT Centre for Research in Intelligent Systems, Monash University, Clayton, Victoria, Australia; University of Leeds, United Kingdom

## Abstract

For many biological applications, a macroscopic (deterministic) treatment of reaction-drift-diffusion systems is insufficient. Instead, one has to properly handle the stochastic nature of the problem and generate true sample paths of the underlying probability distribution. Unfortunately, stochastic algorithms are computationally expensive and, in most cases, the large number of participating particles renders the relevant parameter regimes inaccessible. In an attempt to address this problem we present a genuine stochastic, multi-dimensional algorithm that solves the inhomogeneous, non-linear, drift-diffusion problem on a mesoscopic level. Our method improves on existing implementations in being *multi-dimensional* and handling *inhomogeneous* drift and diffusion. The algorithm is well suited for an implementation on *data-parallel* hardware architectures such as general-purpose graphics processing units (GPUs). We integrate the method into an operator-splitting approach that decouples chemical reactions from the spatial evolution. We demonstrate the validity and applicability of our algorithm with a comprehensive suite of standard test problems that also serve to quantify the numerical accuracy of the method. We provide a freely available, fully functional GPU implementation. Integration into Inchman, a user-friendly web service, that allows researchers to perform parallel simulations of reaction-drift-diffusion systems on GPU clusters is underway.

## Introduction

Complex reaction-diffusion systems, as they appear in the context of biological, chemical and social research, are microscopically governed by Langevin-type stochastic differential equations, where a deterministic process is modulated by random noise [1], [2]. For numerous applications, the assumptions of spatial homogeneity and vanishing drift field cannot be satisfied and need to be relaxed. A large class of complex systems can be described as an ensemble of interacting species where the interaction is modelled by a drift field generated by the individual entities [3]. For instance, a mathematical model of trail formation in pedestrian traffic or ant foraging can be achieved with a Langevin equation that includes various drift terms [4].

A prominent application for a mesoscopic reaction-drift-diffusion approach of the type presented here can be found in molecular biology, more specifically, migration of brain neurons during the developmental stage of the construction of the nervous system in vertebrates [5]. It is well established that cell migration of neurons in the brain is guided by a secreted protein, called Slit [6]. However, experimental data remains ambiguous to the exact nature of its effect on cell motion. In particular, it is unclear whether Slit simply decreases the motility of the migrating cells or if it provides directional guidance cues [5]–[7]. In an attempt to clarify the effects of directional guidance and motility regulation, a compartmentalized random walk model of cell migration, where the transition probabilities between neighboring cells are affected by the presence of an inhibiting or repelling signalling molecule, was developed by Cai *et al.* [8]. The effect of Slit can be easily captured by imposing a state-dependent, spatially inhomogeneous drift-diffusion field on the migrating neurons. In particular, the strength and direction of the guidance field as well as the motility of the neurons are determined by the local density and density gradient of the signalling molecule. We present some preliminary results of this application after the discussion of the test problems below.

To simulate reaction-diffusion models, researchers can choose among a multitude of spatial stochastic solvers. Broadly, one can distinguish between three classes of algorithms with each of them working on a different level of scale. Firstly, *microscopic* methods emphasize the stochastic nature of the problem by focusing on the behavior of individual entities, termed agents [9]. These models track the position and state of each particle individually and therefore provide an exact representation of the underlying problem. Data-parallel implementations of microscopic models can improve runtime performance by two orders of magnitude [10], [11]. The first-passage kinetic Monte Carlo algorithm further improves on this method by introducing disjoint spatial domains (protected zones) where single particles propagate individually and independently until collisions occur [12]–[14]. Needless to say, these algorithms are computationally expensive and are best suited for problems with a low number of individuals, for example, highly diluted solutions. *Mesoscopic* approaches, secondly, sacrifice accuracy for computational speed by discretizing the computational domain into subvolumes. Instead of treating particles individually, these algorithms keep track of the total number of particles of each species per subvolume. Inside each subvolume, reactions can be modelled stochastically by solving the chemical Master equation (CME) [15]–[19]. Diffusion is regarded as transition between subvolumes and is treated either by integrating diffusion terms into the CME [20]–[24] or separately in a stochastic-stochastic hybrid approach [25]–[27]. The later method, also termed operator splitting in the context of applied mathematics [28], is especially suited for implementations on parallel computing architectures [27]. A stochastic-stochastic operator splitting approach based on first-passage time transition rates was presented for pure reaction-diffusion processes without drift on unstructured meshes [29] and extended to include fiber-bound molecular transport in the context of cell physiology [30]. Finally, *macroscopic* algorithms neglect the probabilistic nature of the problem and solve the Fokker-Planck equation for the probability distribution of the particle position, an approach which is only valid if a large number of reacting particles is present [31], [32].

Compartment-based (mesoscopic) stochastic simulation algorithms suffer from the major limitation that they cannot recover the continuous reaction-(drift-)diffusion equation if bimolecular or higher order reactions are involved [33]. Broadly speaking, the problem is that, in the limit of vanishing subvolume size, the reaction probability for bimolecular reactions approaches zero and hence, without renormalization of the reaction rate, the probability density approaches the continuum solution for a freely diffusing particle [34]. Consequently, the subvolume size is bounded from below to guarantee a satisfactory performance of the algorithm. Quantitative bounds are discussed in [33]. For the purpose of this article, however, the operator splitting approach, where reactions and spatial motion are treated separately, allows us to concentrate on numerically solving the drift-diffusion Langevin equation. The integration of reactions into the reaction-diffusion algorithm has been discussed and tested extensively elsewhere [27]. There is no reason to assume that the accuracy of this integration suffers from merely extending the functionality of the diffusion module and, for the sake of readability and to clearly isolate the main results, we choose to omit test problems which explicitly include reactions. A detailed study of this algorithm including reactions will be presented in a future publication.

Generally, stochastic algorithms are computationally expensive and hardware limitations severely restrict their applicability to realistic models and, consequently, parallel implementations are called for [35]. In recent years, graphics processing units (GPUs) have matured sufficiently to provide an accessible hardware platform for general scientific computing in the systems biology community [36]. GPU implementations of spatial stochastic solvers provide tremendous speed ups of up to several orders of magnitude even on standard work station hardware [10], [11], [19], [27].

We present, for the first time, a stochastic algorithm to solve multi-dimensional, inhomogeneous drift-diffusion-reaction problems. While many components of this algorithm have been described previously, no integrated high-performance solution has been presented and evaluated yet. We designed this algorithm as an extension to an existing GPU implementation of a stochastic reaction-diffusion solver [27]. The source code is freely available at http://code.google.com/p/gpgmp/http://code.google.com/p/gpgmp/. We demonstrate the feasibility of our approach with a variety of test cases.

Throughout this article we model microscopic motion as a space-jump process. However, in many biological applications, such as movement of bacteria, the microscopic behavior is mathematically described in terms of a velocity jump process and extensive literature is devoted to the subject [37]–[40]. In this scenario, individual particles change their direction by turning at random, Poissonian-distributed times. Since the direction after the turning event depends on the velocity vector before turning, the positions are now correlated and the random walk looses its Markov property. Provided the correlation time is finite and short with respect to the other time scales involved, we recover an uncorrelated random walk in the long term limit [38], [39]. We will return to this issue in the Methods section.

This article is structured as follows. After briefly introducing the mathematical context and summarizing previous work, we describe our approach in the methods section. In particular, we compare the accuracy of our algorithm with similar methods which are based on discretizing the Fokker-Planck equation. We then provide several test problems which fully explore the capabilities of our implementation in the results section. We conclude with a brief summary of the main results.

## Methods

We aim to solve the general Ito stochastic differential equation (SDE)
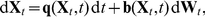
(1)where 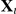 is a stochastic process. Here, 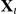 is the position of a particle in space and 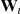 denotes a multi-variable Wiener process. We do not pose any restrictions on the form of the drift and diffusion coefficients, 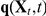 and 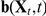, respectively. We will demonstrate below that the algorithm is capable of dealing with general functions. Unlike the algorithm presented in [41]–[43] our approach is readily applicable to multiple dimensions. The implementation we provide, however, is currently restricted to two dimensions and we hope to remove this limitation in a future release. For the purpose of this article, all test problems are simulated on a two-dimensional domain.

An alternative formulation of the same stochastic process can be obtained by computing the conditional probability 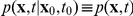 for a particle that is initially located at 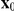 to be found at 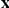 at a later time 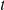. By a transformation of variables in Eq. (1) , one arrives at the Fokker-Planck equation (FPE) for the time evolution of 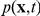 [44]:
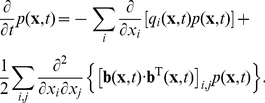
(2)


**Figure 1:**
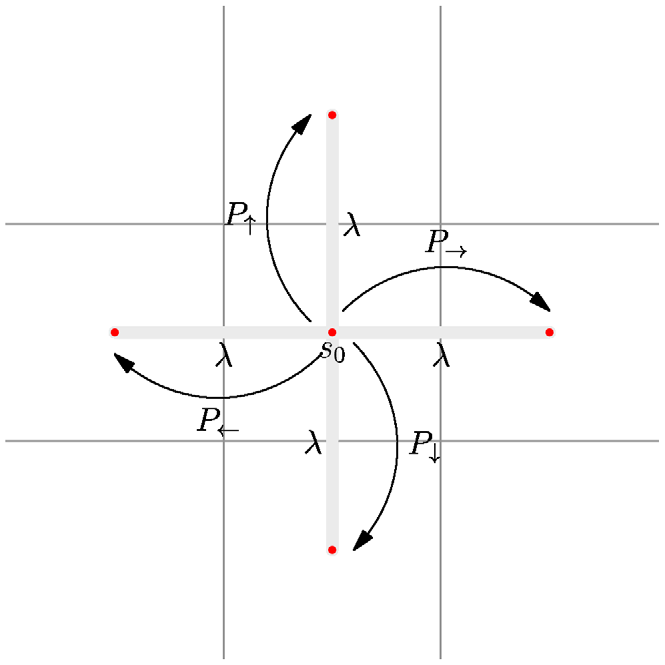
Transition probabilities on a cell-centered grid. The particle jumps to the neighboring grid cells with probabilities 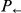, 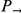, 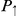 and 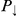. The probability to stay put is given by 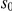.

The mesoscopic approach that we follow here consists of discretizing the computational domain into subvolumes of side length 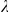 and collectively tracking the number of particles in each subvolume over time. If the diffusivity and drift are smooth functions of 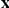, we can obtain an approximate solution by keeping 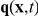 and 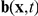 constant inside each subvolume. The drift-diffusion process is then modelled by allowing particles to jump to neighboring cells (note that the multinomial algorithm permits jumps to higher-order neighbors as well [26]) or stay put according to a certain probability distribution ( Fig. 1 ).

The aim of this section is to detail the jump and rest probabilities that correctly reproduce trajectories of Eq. (1) . Owing to the mathematical equivalence of Eqs. (1) and (2) [44], two different approaches present themselves. One possibility is to solve the first-passage time problem for Eq. (1) inside each subvolume to obtain the mean first-passage time and the associated splitting probability [44], [45]. Alternatively, the equivalent Fokker-Planck equation Eq. (2) can be discretized directly and the transition probabilities from the resulting multivariate master equation can be derived [8], [44]. In this work, we present an algorithm which is based on the solution of the first-passage time problem.

Calculating the transition rates by discretizing the corresponding Fokker-Planck equation is straightforward and this will be the starting point of our exposition. The first-passage time algorithm is then described in the following subsection, where we start by briefly recapping the one-dimensional continuous-time and discrete-time algorithms as they were presented in [41]–[43] and then proceed to extend the algorithm to general, inhomogeneous drift-diffusion fields and multiple dimensions. We also compare our method to algorithms based on the discrete FPE formulation. Details about the implementation on graphics-processing units are presented in the last subsection.

### Discrete Fokker-Planck equation

An important class of algorithms, such as the implementation presented in [8], derive the transition probabilities for a particular stochastic differential equation via directly discretizing the equivalent Fokker-Planck equation. For the sake of simplicity, we restrict ourselves here to a one-dimensional problem and note that the multi-dimensional generalization through dimensional splitting, as explained below, is straightforward.

Consider a one-dimensional Fokker-Planck equation with constant diffusivity and drift,
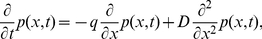
(3)where the diffusivity is defined as 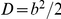. A straightforward discretization with a centered-differencing scheme for 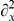 yields the transition probabilities during a time step 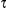 [8],
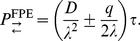
(4)The same numerical scheme can be applied to the more general case of spatially dependent diffusivity and drift and the question arises how the direct discretization method differs from the first-passage time algorithm. We will return to this question below.

In the more general case,
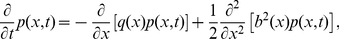
(5)centered-differencing discretization results in a loss of locality for the transition probabilities. For example, [8] consider FPEs of the form
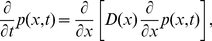
(6)which is equivalent to Eq. (5) if we make the substitution 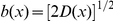 and 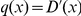. A centered-differencing discretization of Eq. (6) provides the transition probabilities [8]

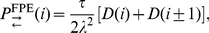
(7)where 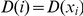 and 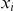 denotes the center of the corresponding grid cell.

These results are well established in the theory of stochastic processes. By means of an expansion in a suitable parameter it can be shown [44] that a master equation with a transition probability to neighboring cells given by
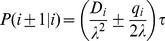
(8)approximates a diffusion-type FPE of the form Eq. (5) *in the limit* 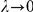.

### First-passage time algorithm

#### Continuous-time random walk algorithm in one dimension

Consider a one-dimensional Ito SDE with globally constant drift and diffusion coefficients (both of these restrictions will be relaxed below):
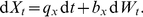
(9)We solve Eq. (9) on an unbounded domain which is discretized into intervals (side length 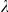) forming a cell-centered grid. The one-dimensional first-passage time problem can be solved analytically for this case [43], [46], [47]. The splitting probabilities (time-integrated jump probabilities) are then [46]

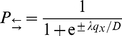
(10)and the exit time probability-distribution function (PDF) is given by
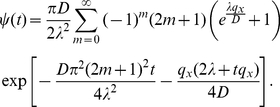
(11)As above, the diffusivity 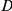 is related to the noise coefficient 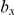 in Eq. (9) through 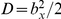.

We can construct a simple algorithm to solve Eq. (9) as follows. Initially, we place a particle at 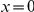. Each time step, we advance the clock by a random increment drawn from the distribution 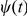 [ Eq. (11) ] and subsequently pick the jump location according to Eq. (10) , where only jumps to the nearest neighbors are permitted. This algorithm constitutes a Montroll-Weiss continuous-time random walk (CTRW) on a lattice. The theory of these models is well understood and we follow the exposition in [48] to compute the first two moments of the displacement.

We start by computing the Laplace transform of the exit time PDF [ Eq. (11) ]:
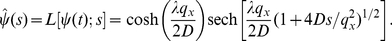
(12)We can expand this expression around 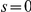,
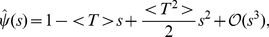
(13)where
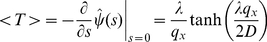
(14)and
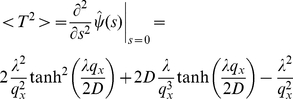
(15)are the familiar expressions for the first two moments of the exit time [46].

It can be shown [48] that the diffusivity and mean velocity of the lattice CTRW are given by
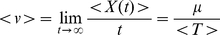
(16)and
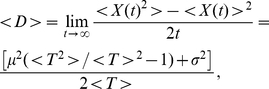
(17)where
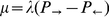
(18)and
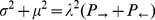
(19)are the mean displacement and mean-square displacement *per step*, respectively. Upon inserting Eqs. (14) , (15) , the according expressions for 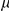 and 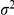 and the transition probability, Eq. (10) , into Eqs. (16) and (17) , we can indeed recover 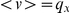 and 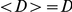.

Eq. (17) is, albeit derived differently, the central result of [43]. For a non-vanishing drift, 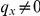, the mean diffusivity depends on the mean jump time as well as on the *variance* of the jump time. Consequently, a naive algorithm with a fixed time step, 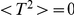, will fail to correctly reproduce the diffusivity. We will see in the next section how an algorithm with a fixed time step can be devised by allowing the particle to stay at rest with a certain probability.

The results presented above are valid for any uncorrelated random walk on a lattice and similar expressions can be derived for correlated random walks [37], [38], [40], [49]. While an *uncorrelated* biased random walk approaches a (parabolic) drift-diffusion equation in the macroscopic limit, the corresponding macroscopic equation for a *correlated* random walk is a hyperbolic equation (the telegraph equation in one dimension or the linear transport equation in higher dimensions) [37], [40]. The fundamental solution of the drift-diffusion equation with constant coefficients is a uniformly moving Gaussian kernel [cf. Eq. (32) below] which clearly exhibits a positive probability density everywhere. In other words, even for 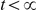, the particle has a non-vanishing (albeit small) probability to be found anywhere in the infinite domain. This is a consequence of the infinite signal propagation speed for parabolic equations. Hyperbolic equations, on the other hand, do not show this unphysical behavior and it can be demonstrated that, in the long term limit, any influence of short-term correlations is lost and the solution approaches the limiting solution of a diffusion equation. Whether short-term correlations can be safely neglected strongly depends on the system in question. We note, however, that many biological applications allow a description as a Markov random process [44].

We finally remark that the basic model of a biased random walk can be extended in several ways. A comprehensive, comparative review of alternative models, such as anomalous diffusion and velocity jump random walk models can be found in [40].

#### Discrete-time random walk

[43] demonstrate how to construct a fixed time step algorithm that correctly reproduces the diffusivity and mean velocity. The formalism established in the previous subsection allows an alternative derivation of these results which we present here. To this end, the particle is allowed to stay at rest with a probability 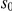 during each time step. The probability to move after exactly 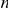 time steps is then 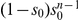 and the jump time PDF can be written as
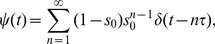
(20)where 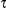 is the duration of the fixed time step and 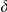 denotes the Dirac delta function. The Laplace transform is easily computed and, just as in the previous section, can be expanded around 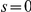:
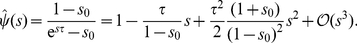
(21)A comparison of the coefficients with Eq. (13) reveals that the choice
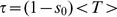
(22)and
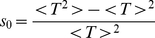
(23)yields the equivalent discrete-time random walk. This constitutes the fixed time step algorithm for highly biased random walks as devised by [43].

#### Dimensional splitting

The fixed time step algorithm described in the previous subsection can be extended to two dimensions under the restriction that the drift field is parallel to one coordinate axis [43]. Here, we show how to relax this restriction and present a general multi-dimensional algorithm.

The key idea for solving the two-dimensional problem is to treat jumps in 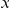 and 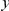 direction separately, an approach termed *dimensional splitting* in the context of numerical algorithms for deterministic PDEs. That is, instead of having one diffusion sweep for both directions, we have two separate one-dimensional sweeps for each direction. The benefit of this approach is that we can use the formalism from the previous subsection which ensures that both, the diffusion constant and the velocity, are reproduced correctly. When applied to deterministic PDEs, dimensional splitting is known to be second-order accurate only if 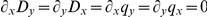, where we assume that the diffusivity matrix is diagonal [50]. Moreover, the operator splitting approach used to integrate reactions will be second-order accurate only if the velocity field is divergence free and the reaction rates are homogeneous. Generally, neither of these assumptions holds and we therefore expect a first-order splitting error. However, it is not obvious how these considerations transfer to a stochastic algorithm. We therefore restrict ourselves to quantify the numerical error of our implementation with suitable test problems.

The transition probabilities for one-dimensional left/right transitions are given by Eq. (10) , the survival probability is computed according to Eq. (23) and the sweep times are set by Eq. (22) . The mean and variance of the transition time are given by Eq. (14) and Eq. (15) . Written in pseudo code, the algorithm looks as follows:

Compute 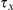 and 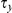 [from Eq. (22) ].

Initialize global time 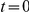 and set alarm times 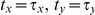.

**while** 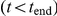


 **if** 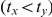


  Perform 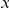 sweep.

  Set global time 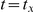. Set next alarm time 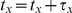.

 **elseif** 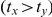


  Perform 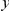 sweep.

  Set global time 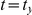. Set next alarm time 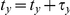.

 **elseif** 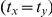


  Perform 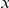 sweep.

  Perform 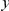 sweep.

  Set global time 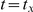. Set next alarm time 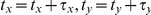.

 **endif**

**endwhile**

The sweeps consist of redistributing the available particles in each cell to the neighboring cells. Each particle can either rest [with probability 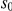 from Eq. (23) ] or jump [with probabilities 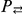 given by Eq. (10) ]. The pseudo code for the diffusion sweep looks as follows:

**forall** cells **do**

 **forall** particles in cell **do**

  Draw random number 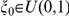.

  **if** 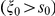


   Draw random number 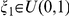.

   **if** 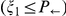 jump left.

   **else** jump right.

  **endif**

 **enddo** particles

**enddo** cells

#### Inhomogeneous drift-diffusion

While the two-dimensional algorithm works well if 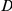 and 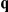 are constant over the whole integration domain, we need to extend it to incorporate inhomogeneous drift and diffusivity. The main issue on the algorithmic side is that the time step 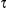, as defined by Eqs. (22) and (14) , now implicitly depends on the cell location through 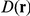 and 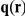. On the other hand, a GPU implementation requires a common global time step to avoid synchronization issues. This is because the highly-specialized graphics card architecture only allows very restricted communication between individual cells. We elaborate on the particular GPU architecture and the problems associated with it in the implementation section below.

A simple approach to inhomogeneous problems is to compute the time step 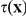 for each cell, find the minimum 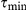 over the whole domain, set 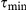 as the common global time step and scale the transition and rest probabilities for each cell accordingly. For the convenience of the reader, we first collect the important equations.

From Eqs. (16) , (17) [along with the definition of 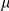 and 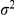, Eqs. (18) and (19) ], and (21) , we can compute the macroscopic drift and diffusivity for any lattice random walk with a fixed time step 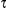 as a function of the transition probabilities 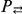 and the rest probability 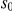:
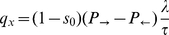
(24)and
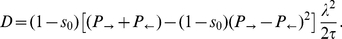
(25)Note that 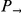 and 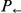 are the conditional probabilities for a transition to a neighboring cell, provided that the particle actually jumps (rather than stay at rest during the time step). Hence, we have the additional requirement
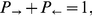
(26)which algebraically closes the system. We can solve (24)–(26) for 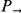, 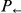, and 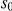, finding:
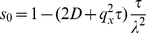
(27)and
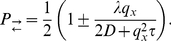
(28)


Eqs. (27) and (28) allow us to compute the transition and rest probabilities for an arbitrary time step 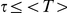, where 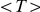 is defined by the one-dimensional first-passage problem [cf. Eq. (14) ]. Indeed, if we plug in the canonical time step [Eqs. (22)–(23) ] [43],
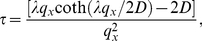
(29)we can recover the splitting probabilities Eq. (10) . A one-dimensional implementation in pseudo code is presented below. The extension to multiple dimensions via dimensional splitting is obvious.

Compute 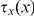.

Find 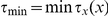.

Initialize global time 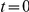 and set alarm time 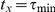


**while** 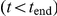


 Compute 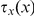.

 Find 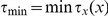.

 Perform 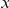 sweep with global time step 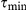 and probabilities 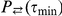 and 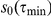.

 Set global time 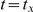. Set next alarm time 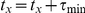.

**endwhile**

How do the transition probabilities derived here compare to directly discretizing the corresponding FPE? The connection becomes obvious if we write down the Taylor expansion of the unconditional transition probabilities 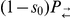, with 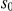 and 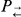 given by Eqs. (27) and (28) , respectively. We find
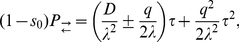
(30)and confirm that Eq. (4) provides a first-order approximation to the complete transition probabilities. The missing second-order contribution 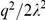 naturally corresponds to the variance of the jump time and vanishes for 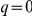. As we will see later, neglecting this second-order contribution leads to higher inaccuracies if the problem in question is drift dominated.

### Implementation

We implement the algorithm described above as an extension to an existing program package, gpgmp [27]. gpgmp is a mesoscopic, stochastic solver for *homogeneous* reaction-diffusion problems and separately treats reactions and diffusion in an operator-splitting fashion. This modular design allows us to easily exchange the homogeneous diffusion module with these extensions while leaving the reaction module untouched.

The GPU implementation of the inhomogeneous drift-diffusion module closely follows the design of its homogeneous equivalent [27]. The computational domain 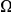 is divided into equally spaced cubical subvolumes with common side length 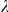. Currently gpgmp and the deterministic solver module only support two-dimensional domains. Each cell is assigned to exactly one thread on the GPU. They are executed in parallel. In order to reduce memory access overhead, the GPU architecture groups two-dimensional units of threads into *blocks*, which operate independently. Global synchronization across block boundaries is not permitted and hence can only be achieved by returning control to the host CPU. We omit a detailed description of the reaction algorithm here as it has been described elsewhere [27].

The main loop is executed on the host CPU and is responsible for calling the various GPU kernels. Whenever global synchronization across block boundaries is required, program control is returned to the host. The main responsibility of the outermost loop is to handle dimensional splitting for all species involved. This is done by sorting the diffusion events for each species and each direction in a global time line. Whenever the simulation encounters a diffusion event, the corresponding species is diffused and the next diffusion time (for the species and direction in question) is computed.

**forall** species 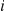


 Compute 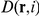 and 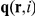 in kernel computeDiffusionConstants.

 Compute time step in 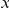 direction 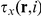 [ Eq. (29) ] in kernel computeIndividualTimestep.

 Reduce over blocks and find minimum 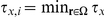 in kernel reduceBlocks.

 Compute time step in 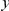 direction 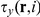 [ Eq. (29) ] in kernel computeIndividualTimestep.

 Reduce over blocks and find minimum 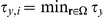 in kernel reduceBlocks.

**endfor**

Find first diffusion event time 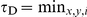.

**while** 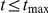


 **if** next event is 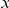 diffusion for species 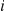


  Perform 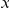 sweep for species 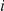 according to probabilities (27)–(28) for 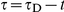 in kernel diffuse

  Update particle count over block boundaries in kernel updateState.

  Compute 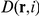 and 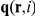 in kernel computeDiffusionConstants.

  Compute time step in 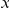 direction 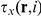 [ Eq. (29) ] in kernel computeIndividualTimestep.

  Reduce over blocks and find minimum 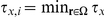 in kernel reduceBlocks.

 **endif**

 **if** next event is 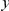 diffusion for species 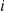


  Perform 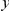 sweep for species 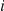 according to probabilities (27)–(28) for 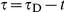 in kernel diffuse

  Update particle count over block boundaries in kernel updateState.

  Compute 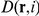 and 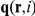 in kernel computeDiffusionConstants.

  Compute time step in 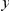 direction 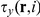 [ Eq. (29) ] in kernel computeIndividualTimestep.

  Reduce over blocks and find minimum 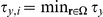 in kernel reduceBlocks.

 **endif**

 Set global time 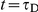.

 Find next diffusion event time 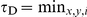.

**endwhile**

The implementation of the various kernels is straightforward. With the exception of the reduceBlocks kernel, each thread works on exactly one subvolume. The computeDiffusionConstants routine computes the diffusivity and drift for each cell according to the specific problem. computeIndividualTimestep calculates the time step [ Eq. (29) ] for each species (and direction) in the particular subvolume. In order to find the global minimum time step of the whole domain (“reduce” over all blocks), we implement a standard parallel scan algorithm which requires two sweep phases [51]. The up-sweep phase of the parallel scan is performed in the computeIndividualTimestep kernel, while the reduceBlocks kernel is responsible for the down-sweep phase. The diffusion kernel works exactly as its homogeneous counterpart [27] except that the transition probabilities are computed according to Eqs. (27)–(28) . We then perform a random experiment for each particle of the species in question in the subvolume to determine if and where it moves. Finally, we need to update the particles at the block boundaries in updateState.

## Results

In this section, we are concerned with the accuracy of our implementation. It is sufficient for our purpose to test the diffusion module separately. The integration with the Gillespie algorithm that performs reactions has been described elsewhere [27] and remains unchanged in our implementation.

We performed comprehensive tests trying to encompass the most common situations. All simulations are set up on a two-dimensional, square grid with side length 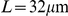, with varying granularity 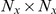, where 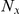 denotes the number of subvolumes per dimension. The cell-centered physical coordinate system is mapped to the subvolume index 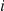 by a linear transformation, viz.
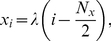
(31)such that the origin is located at the center subvolume 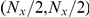.

We start with a simple two-dimensional homogeneous drift-diffusion problem and progressively add more functionality to the tests by implementing a geometric Brownian motion problem (to test inhomogeneous diffusivity and drift), a two-dimensional Ornstein-Uhlenbeck process (which demonstrates the validity of dimensional splitting) and a fully non-linear problem. We conclude this section with a biological application where we model the influence of the signalling molecule Slit on migrating neurons.

### Homogeneous biased diffusion

We set up a test problem with a globally homogeneous diffusivity and drift, i.e. 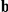 and 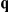 are constant over the whole domain. We assume 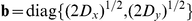. Initially a number 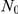 of particles is located in the center at 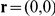. It is straightforward to show that the solution of the corresponding Fokker-Planck equation at time 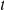 is then given by
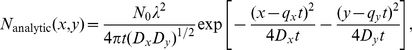
(32)where 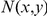 denotes the number of particles in the cell centered at 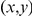.

The analytic solution, Eq. (32) , allows us to obtain a quantitative estimate for the accuracy of the simulation via the root-mean square error (RMSE),
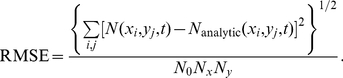
(33)


We distribute 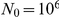 particles over the whole domain which is divided into 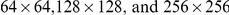 lattice cells, respectively. The diffusivity is 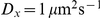 and 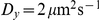 and the drift field is given by 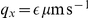 and 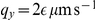, where 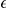 is varied from 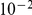 to 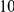. Each simulation is carried out 100 times and we average over all runs. We chose outflow boundary conditions and stop each run at 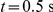 to ensure that boundary effects are excluded.

**Figure 2:**
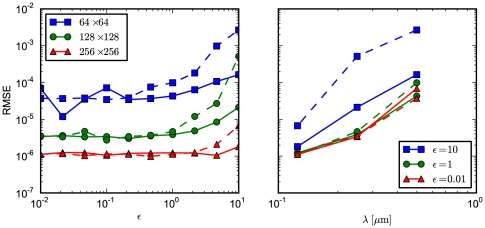
RMSE for the biased diffusion test problem. The root mean-square error of the simulation output is calculated relative to the analytic solution, with 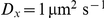 and 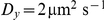 at 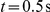. The solid curves indicate results from simulations which were done with transition probabilities computed from the FPT. The dashed curves, in contrast, displays the RMSE from simulations based on a discretization of the FPE. In both cases, 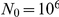 molecules are located in the center subvolume initially. (left) We display the RMSE as a function of 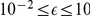 for 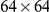 (blue squares), 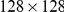 (green circles), and 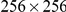 (red triangles) subvolumes. (right) Shown is the RMSE as a function of subvolume side length 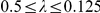 for 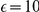 (blue squares), 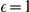 (green circles), and 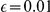 (red triangles).

The results are presented in Fig. 2 (left panel) which displays the RMSE for 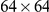 (blue), 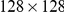 (green), and 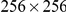 (red) subvolumes. We compute results for simulations where the transition probabilities are derived from the first-passage-time problem (solid curves) and from a discretization of the Fokker-Planck equation (dashed curves). In both cases, the accuracy improves with a finer granularity. Overall, the code performs satisfactory, with a slight tendency for the accuracy to worsen in the drift-dominated (high 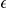) set up. As expected, the FPE-based implementation performs worse for high 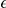, i.e. if the problem is drift dominated. For comparison, we display the results for fixed drift contributions 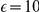 (blue), 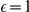 (green), and 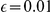 (red) and varying grid spacing 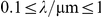 in the right panel of the figure.

We compare our simulations with recent results achieved for uncorrelated, biased, space-continuous random walks with varying speed [52]. These authors consider a random walker who, at the end of each step, changes the speed and direction according to a general distribution. It can be shown that, in the long term limit, this particular model results in a drift-diffusion equation, where the drift and diffusion coefficients are determined by the distribution of the velocity changes [52]. An important result of this work is the observation that diffusion in this case is typically anisotropic, where the component of the diffusion tensor along the preferred direction is smaller than the perpendicular contribution if the speed of the random walker is fixed and larger in the opposite case.

### Geometric Brownian motion

We implement this test problem to assess the capability of the code to handle inhomogeneous diffusivity and drift. The geometric Brownian motion (GBM) process is defined by the SDE
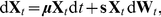
(34)with 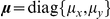 and 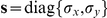 diagonal matrices which are held constant over the whole domain.

It can be shown that the corresponding FPE prescribes a log-normal type PDF,
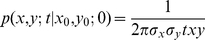
(35)

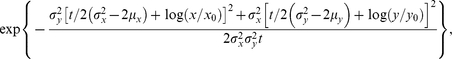
that allows us to compute the RMSE for our simulation outputs.

In order to avoid the pathological case of vanishing diffusivity in the center subvolume, we shift the origin of the coordinate system by the width 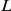 of the domain, i.e. the coordinate system is given by
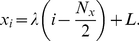
(36)


**Figure 3:**
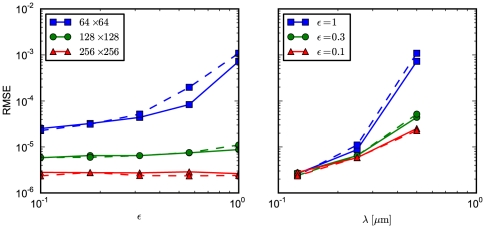
RMSE for the geometric Brownian motion test problem. The root mean-square error of the simulation output is calculated relative to the analytic solution as a function of the drift field contribution 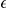 (left panel) and the subvolume side length 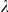 (right panel). Initially 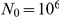 molecules are distributed at 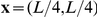. (left) Shown are results for 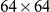 (blue squares), 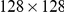 (green circles) and 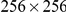 (red triangles) subvolumes. We vary 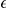 over 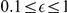. (right) We display the RMSE for 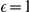 (blue squares), 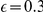 (green circles), and 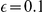 (red triangles), where 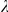 is varied over 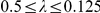.

We simulate the multiplicative noise problem with initially 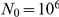 particles located at 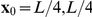. The diffusivity coefficient is held constant at 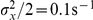 and 
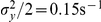
. We vary the drift field coefficient 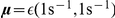 over 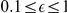. The results are presented in Fig. 3 (left panel) which shows the RMSE of the simulation output. We compute solutions with different granularities, where the number of subvolumes varies from 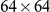 (blue curves) over 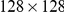 (green curves) to 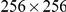 (red curves), for an FPT (solid curve) and an FPE (dashed curve) algorithm. The relative error is acceptable and generally increases along with increasing contribution of the drift field. The benefit of a higher granularity on the accuracy of the solution is obvious and can be ascribed to the zeroth-order approximation of the inhomogeneous fields. Finally, the FPE algorithm (dashed) curve performs similarly well as its FPT counterpart (dashed curve). For comparison, we include results for fixed drift contributions 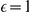 (blue), 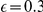 (green), and 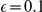 (red) and varying grid spacing 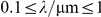 in the right panel of the figure.

### Ornstein-Uhlenbeck

In order to assess the accuracy of the dimensional splitting approach, we implement an Ornstein-Uhlenbeck process, which is an ordinary Wiener process amended by a drift term. It can be described by the stochastic differential equation
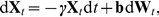
(37)where 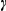 is a constant matrix (not necessarily diagonal) and 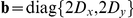 encodes the diffusivity. The theory of Ornstein-Uhlenbeck processes is well understood [44], [53] and the time evolution of the mean vector and covariance matrix are known to be
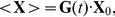
(38)and
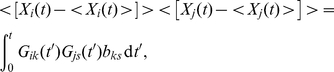
(39)respectively. Here, the Green's function 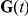 is given by the matrix exponential 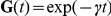. Integration of Eq. (39) is straightforward and the corresponding PDF is a Gaussian distribution,
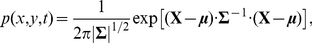
(40)with the mean 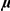 and covariance matrix 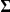 given by (38) and (39). This expression provides us with the means to quantify the accuracy of our implementation for a process involving an inhomogeneous drift field.

**Figure 4:**
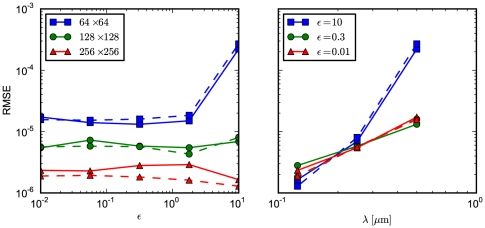
RMSE for the Ornstein-Uhlenbeck test problem. The root mean-square error of the simulation output is calculated relative to the analytic solution. Initially 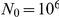 molecules are distributed at 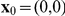. Simulations are run with the FPT implementation (solid curve) and the FPE implementation (dashed curve). (left) Shown are results for 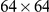 (blue squares), 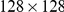 (green circles) and 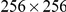 (red triangles) subvolumes. We vary the drift field parameter over 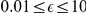. (right) We display the RMSE for 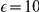 (blue squares), 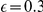 (green circles), and 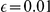 (red triangles), where 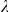 is varied over 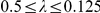.

We set up a simulation with 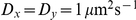 and 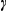 given by 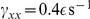, 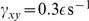, 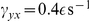, and 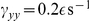, where we vary the parameter 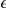 over 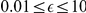. Initially, 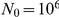 particles are located at the center subvolume, which constitutes the origin of the coordinate system. We then compute the RMSE from the simulation output (averaged over 100 runs) and the theoretical solution Eqs. (38)–(40) . The results are presented in Fig. 4 (left panel), which displays the RMSE as a function of 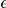. We perform simulations for different granularities, viz. 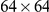 (blue curves) over 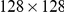 (green curves) to 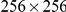 (red curves), where we distinguish between an FPT implementation (solid curve) and an FPE algorithm (dashed curve). For comparison, we display the results for fixed drift contributions 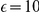 (blue), 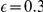 (green), and 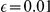 (red) and varying grid spacing 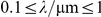 in the right panel of the figure.

The accuracy of all simulations improves with finer granularity. If the problem is drift-dominated (high 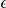), the accuracy worsens for a coarse granularity. There is no clear trend when comparing the FPE implementation (solid curve) to the FPT implementation (dashed curve). We attribute this behavior to the fact that the numerical noise is dominated by the poor (zero-th order) approximation of the inhomogeneous drift field for this test problem.

### Non-linear

We conclude the test series presented here with a genuine nonlinear benchmark model, which has been used previously to assess the accuracy of algorithms for solving the nonlinear, time-dependent Fokker-Planck equation [54], [55]. The time evolution of this test problem is governed by the one-dimensional, nonlinear SDE
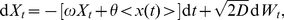
(41)where the drift field implicitly depends on the probability distribution 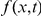 through its first moment
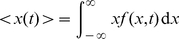
(42)and the particle is initially located at 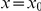. An analytic solution for the FPE corresponding to Eq. (41) can be constructed, viz. [55]

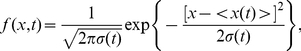
(43)where the moments are given by
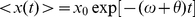
(44)and
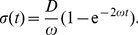
(45)


**Figure 5:**
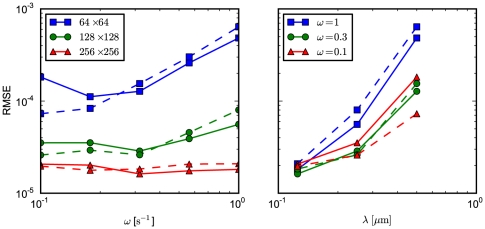
RMSE for the non-linear test problem. The root mean-square error of the simulation output is calculated relative to the analytic solution as a function of the diffusivity 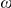 (left panel) and subvolume side length 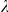 (right panel) for the nonlinear test problem. We display results for an FTP implementation (solid curve) and the FPE counterpart (dashed curve). Initially, 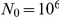 molecules are distributed at 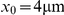. (left) We plot the RMSE for 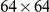 (blue squares), 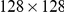 (green circles) and 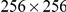 (red triangles) subvolumes and vary the diffusivity over 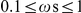. (right) We display results for 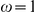 (blue squares), 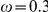 (green circles), and 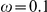 (red triangles), where 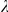 is varied over 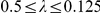.

This solution allows us to evaluate the accuracy of our implementation by computing the RMSE between our simulation output and the analytical solution Eqs. (43)–(45) . Similar to the previous test problems, we set up a model where the drift field and diffusivity is given according to Eq. (41) . Since the drift component is implicitly time dependent, it needs to be evaluated after each diffusion time step. For this purpose, we provide an additional GPU kernel which computes the first moment 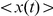 from the current particle state. We initialize the simulation with 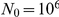 particles located at 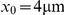. We shift the coordinate system by an amount 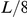, i.e.
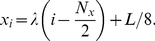
(46)We fix the parameters 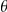 at 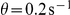 and vary 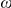 over 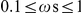. We run all simulations until the system approaches the asymptotic state, i.e. the maximum runtime is given by 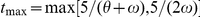. We average over 100 runs and compare the output to the analytical solution. Fig. 5 (left panel) displays the RMSE as a function of 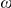 for several granularities. Shown are results for 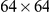 (blue curves), 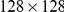 (green curves) and 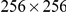 (red curves) subvolumes, where we distinguish between the FTP (solid curve) and FPE (dashed curve) algorithms. For comparison, we display the results for fixed drift contributions 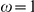 (blue), 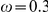 (green), and 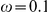 (red) and varying grid spacing 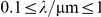 in the right panel of the figure.

The accuracy improves with higher granularity. For 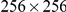 subvolumes (red curves), the accuracy of the code is comparable to the accuracy of a (deterministic) method to solve the non-linear FPE based on distributed approximating functionals [54], [55]. For lower granularities (blue and green curves), a slight trend for the accuracy to worsen with increasing 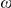 is perceivable. The FPE implementation tends to perform slightly worse than the FPT algorithm, in particular for higher 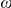.

### A biological application: cell migration of neurons

We conclude our discussion with an example from the field of mathematical biology that serves to illustrate a biological problem that requires inhomogeneous, state-dependent, drift-diffusion. It also underlines the increasing importance of a synergetic interplay between experiment and computer simulations to unequivocally interpret seemingly ambiguous experimental results. We note that the purpose of this presentation is merely illustrative. The aim of this subsection is to reproduce literature results in the framework of our algorithm. We will present comprehensive results in an upcoming publication.

The question we are addressing here concerns the influence of the signalling molecule Slit on cell migration of neurons in the brain. A signalling molecule can affect the motility of the migrating cell, the direction of its motion or a combination of both. Motility regulators are divided into *inducers* or *inhibitors* depending on whether they promote or reduce the cell motility. Characteristic for motility regulators is that they can be effective even if they are present in a constant concentration. If, on the other hand, the signalling molecule provides directional guidance cues it is termed either an *attractant* or a *repellent*. With these molecules, the directional information is encoded in the concentration gradient with attractants imposing a motion along the gradient, i.e. towards the source, onto the migrating molecules while repellents cause the cells to move towards a lower concentration of the repellent [8]. Whether a particular substance acts as motility regulator or provides directional guidance is often difficult to decide experimentally.

Early experiments provided evidence for a repellent [6] as well as inhibiting [5] effect of Slit on migrating neurons. In an attempt to resolve the ambiguity, Ward *et al.* designed a time-delayed experimental setup [7]. A circular explant from postnatal rat brains serves as a source of migrating neurons. Without an application of Slit, these neurons propagate symmetrically over time. After 24 h, an aggregate of Slit was placed at one edge of the domain which provides a steady concentration gradient of the signalling molecules. After another 24 h passed, the spatial distribution of neurons was observed to be clearly skewed away from the Slit source. Furthermore, instead of aggregating at some point between the Slit source and the explant (as would be expected if Slit had a purely inhibiting effect) the neurons moved *away* from the Slit aggregate. This experiment provided conclusive evidence for the assumption that Slit is a repellent of neurons. Complementing these results, Cai *et al.* performed Monte-Carlo simulations of the experimental set up [8]. The particular focus of their work was the question if data of population-level cell distributions and individual-level cell movement would allow to draw conclusions about the underlying microscopic behavior. In this section, we reproduce their main results using our computational framework.

Cell migration on a microscopic level is typically modelled as a continuous-time, discrete-space Markov process [56]–[60] and is hence well suited for the approach presented here. Following [56], [57], we start with a continuous-space stochastic differential equation of the form Eq. (1) where 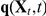 and 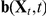 are now termed the chemotaxis coefficient and motility, respectively. During migration, the cell can interact with environmental cues and various cell-sensing strategies have been discussed in the literature [58], [59]. The nature of the interaction can be either *strictly local*, where only local field variables are considered, *neighbour based*, i.e., the cell makes “decisions” based on field variables in the neighboring subvolumes, *local average*, where the interaction is modelled as some sort of average between neighboring subvolumes, or *gradient based*, where the cell responds to gradients of field variables. We choose a variant of the local average model which encapsulates the interactions in a single, state-dependent, scalar field variable 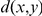 and write down the two-dimensional SDE
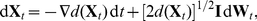
(47)where 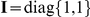 denotes the two-dimensional identity matrix and we compute the spatial derivatives of 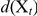 with a standard centered-difference. The corresponding master equation can then be derived by computing the transition probabilities (4) or (28) . Using the limiting procedure described in [58], [59] we can easily recover the macroscopic FPE
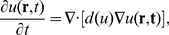
(48)which corresponds to a classical (minimal) Keller-Segel model with vanishing chemotactic sensitivity [56].

The behavior of a migrating cell in our model is determined by (i) cell-cell interactions and (ii) interactions with a signalling molecule. Following [8], we adapt the *contact inhibition of cell locomotion model* [61], [62] in the simple form
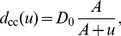
(49)where 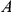 is a saturation parameter and 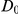 denotes the motility of a single cell. We then incorporate the interaction of neurons with the signalling molecule Slit as an exponential function
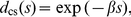
(50)where 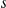 denotes the Slit concentration. After application at 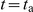, the distribution of Slit quickly attains a steady state [8] and we can simplify
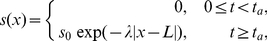
(51)where we choose 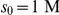 and treat the scale length 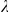, which is determined by the diffusivity of Slit and the source strength, as a free parameter. We assume that slit is applied at the edge of the computational domain (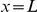). We finally combine the interaction terms (49) and (50) into a single expression
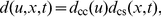
(52)where we emphasize the explicit spatial and temporal dependence and note that the quantity 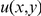 is evaluated in each subvolume through 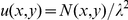 where 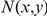 denotes the number of particles in that particular subvolume and 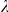 is the grid spacing.

**Figure 6:**
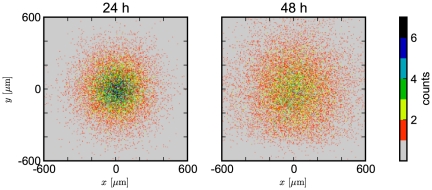
Density map of the cell distribution for a cell migration simulation. We show the number of cells per subvolume for unregulated migration (left) and after placement of the signalling molecule Slit at the bottom edge of the domain (right). The Slit aggregate is placed at 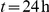 and the repelling effect on the migrating cells is clearly visible.

Eq. (47) in conjunction with Eqs. (49)–(52) provides all the ingredients to reproduce the experiment presented in [7]. For convenience, we collect all model parameters in Table 1. We start the simulation by placing a circular explant of neurons (diameter 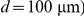 at the origin and let the cells migrate freely, i.e. restricted only to the contact-inhibition cell-cell interaction. The left panel of Fig. 6 displays the copy count of neurons per subvolume at 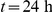. As expected, the cells are symmetrically distributed in a manner similar to uninhibited diffusion. At 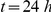, we add the Slit aggregate at the bottom edge of the computational domain and let the simulation proceed for another 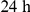. The spatial cell distribution is presented in the right panel of Fig. 6 . We clearly observe how neurons avoid the bottom part of the domain and, more importantly, that some neurons have reverted direction and migrated away from the Slit source. The latter observation is characteristic for the repellent character of Slit as it was observed in the experiment [7], [8].

**Table 1:** Simulation parameters for the cell migration model.

Parameter
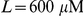
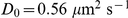
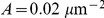
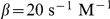
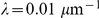
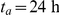

Simulation parameters for the cell migration model Eqs. (47) and (49)–(52) .

## Discussion

In this article, we present, for the first time, a stochastic, mesoscopic, cellular-automaton type algorithm that, based on solutions of the first-passage time problem inside subvolumes, computes sample paths of the corresponding probability distribution function for inhomogeneous, non-linear, drift-diffusion problems. In conjunction with an operator-splitting approach for separating reaction chemistry from spatial motion [25], [27], our implementation provides a powerful tool to compute stochastic solutions to fully non-linear, reaction-drift-diffusion problems.

The contributions of the present approach are three-fold. (i) We extend the first-passage time algorithm [43] to higher dimensions without restrictions on the drift field. (ii) We allow inhomogeneous, non-linear, drift-diffusion fields. (iii) We present a data-parallel implementation on GPUs which allows for a considerable performance gain. To the best of our knowledge, this has not been achieved before. Existing stochastic algorithms either neglect drift fields altogether [23]–[25], [63], severely restrict their applicability to coordinate-axis aligned fields [43] or are optimized for a low particle number approximation [12]–[14].

In this article, we introduce a method that is based on a *microscopic* description of the particle dynamics. We start with a stochastic differential equation and compute the transition rates for the mesoscopic algorithm from the corresponding first-passage times. This approach correctly reproduces the diffusivity and drift coefficients on a discrete mesh. In contrast, an approach based on discretizing the *macroscopic* Fokker-Planck equation looses accuracy in the limit of small subvolume size. Apart from being conceptually more satisfying, our method hence proves to be more accurate for coarse grids and high drift fields.

We demonstrate the validity of our approach with a variety of test problems where an analytic solution is available. The accuracy of our implementation matches, or outperforms, the accuracy of existing macroscopic solvers. An obvious improvement of our algorithm can be attained by relaxing the assumption of constant diffusivity and drift inside each subvolume. Instead, one could imagine to allow a linear (or higher order) spatial variation of the drift and diffusion coefficients in order to approximate the global inhomogeneous field by a piecewise-linear function. It is then possible to derive the transition probabilities by solving the first-passage-time problem for an Ornstein-Uhlenbeck process. This task seems feasible, albeit not trivial, and we plan to enhance the implementation accordingly in future work.

A main objective of this project is to provide researchers with “barrier-free” access to high-performance computational resources for large-scale stochastic modelling. Our current effort hence focuses on integrating the algorithm at hand into Inchman, a convenient easy-to-use web interface to gpgmp (available at http://www.csse.monash.edu.au/~berndm/inchman/). Inchman allows users to upload chemical reaction networks, specified in the popular systems-biology markup language (SBML), amend them with information about the spatial structure and run large-scale simulations. Inchman builds on the Nimrod toolkit (http://www.messagelab.monash.edu.au/Nimrod) and hence allows versatile parameter exploration, such as parameter sweeps [64].
